# Probabilistic Evaluation of 3D Surfaces Using Statistical Shape Models (SSM)

**DOI:** 10.3390/s20226554

**Published:** 2020-11-17

**Authors:** Javier Pérez, Jose-Luis Guardiola, Alberto J. Perez, Juan-Carlos Perez-Cortes

**Affiliations:** Instituto Tecnológico de Informática (ITI), Universitat Politècnica de València, 46022 Valencia, Spain; javierperez@iti.es (J.P.); joguagar@iti.es (J.-L.G.); aperez@iti.es (A.J.P.)

**Keywords:** 3D surface evaluation, 3D reconstruction, statistical shape model, quality assessment, 3D metrics

## Abstract

Inspecting a 3D object which shape has elastic manufacturing tolerances in order to find defects is a challenging and time-consuming task. This task usually involves humans, either in the specification stage followed by some automatic measurements, or in other points along the process. Even when a detailed inspection is performed, the measurements are limited to a few dimensions instead of a complete examination of the object. In this work, a probabilistic method to evaluate 3D surfaces is presented. This algorithm relies on a training stage to learn the shape of the object building a statistical shape model. Making use of this model, any inspected object can be evaluated obtaining a probability that the whole object or any of its dimensions are compatible with the model, thus allowing to easily find defective objects. Results in simulated and real environments are presented and compared to two different alternatives.

## 1. Introduction

Industrial inspection is an important activity which requires fast and accurate systems. Many inspection processes are still human labour intensive. While the metrology labs in industry routinely make use of advanced 3D metrology equipment, production floors often lack the technology to automatically inspect and assure the quality of the production. As a consequence, the manufactured objects must be sampled and moved to the lab so a skilled worker who can operate the laboratory equipment can measure them. This process is time consuming and also increases production costs requiring a specialized person to measure and interpret the results. The complexity of the designed objects rises as the manufacturing technologies improve, and the economic margins, at the same time, are decreasing due to global competition.

Furthermore, before these complex objects are assembled into a product, many measurements must be performed to assure that the final product is fully functional and there are no deviations or small errors that can cause defects. To obtain 100% of the production inspected, the inspection system must be placed in line, so it can automatically check all the objects produced.

Although many objects require a 3D inspection to assure its quality, typical inspection systems in industry are still 2D or 2.5D, leaving hidden regions. 3D systems that are able to capture a larger proportion of the object surface usually require long processes with sequential operations using stereoscopy, laser beams or structured light [1,2,3]. Other systems make use of robotic arms to move the object in front of a sensor such as Fei et al. [4] or Brosed et al. [5]. It is also possible to obtain the 3D reconstruction from multiple views as Perez-Cortes et al. [6] show. In Bi et al. [7] a survey of non-contact 3D scanners with a classification of their technologies from a manufacturing perspective is presented. More recent techniques include deep learning from single image [8], using RGB-D cameras [9] or using histology sensors [10].

Regardless of the acquisition technique, once a 3D object is acquired and reconstructed, it is usually necessary to detect the errors and differences against a reference or a model. In that way, at the end of the evaluation, it is possible to decide if the object has errors that will potentially cause a defect in the final product, and discard it if necessary. This is even more challenging in the case of objects which shape has elastic manufacturing tolerances. Elastic manufacturing tolerance can be defined as a continuous range of shape limits for accepting an object that must be described as an elastic deformation of this object. This restriction applies only to the acceptation limits, not to the physical properties of any sample of that object.

**Figure 1:**
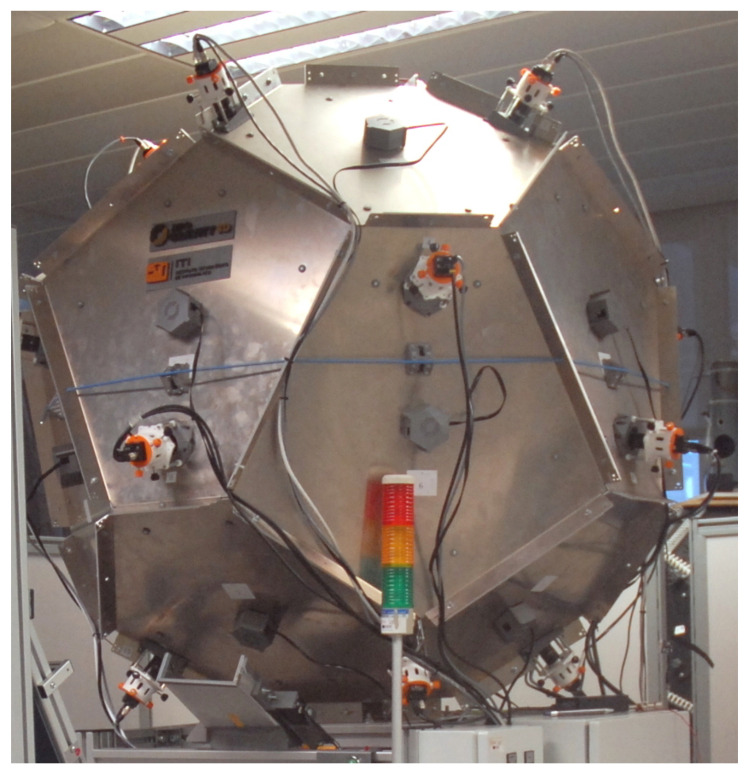
Industrial inspection system Zero Gravity 3D (ZG3D).

Although the results of this work are generalizable, the experiments presented use a 3D reconstruction technique from multiple silhouettes as explained in Perez-Cortes et al. [6] and 3D models acquired with a ZG3D patented device [11] as the one shown in Figure 1.

The work is structured as follows. Section 2 describes the current state of the art in 3D surface comparison and SSMs. Then, the training and evaluation stages of the proposed algorithm are described in Section 3 and Section 4 respectively. Methodology assessment is discussed in Section 5. Proposed algorithm is compared with two industrial inspection solutions in Section 6, showing simulated and real results with a ZG3D device. Finally, conclusions and future work are presented in Section 7.

## 2. State of the Art

Industrial inspection operation has been traditionally performed using Coordinate Measuring Machines (CMM). These devices take physical measurements at different points of interest at the target object, improving the efficiency of inspection processes. However, as they are making point contact with the object at each sampled point, they are time-consuming so the number of measurements is limited. They can also distort the surfaces of elastic or delicate materials and require expert knowledge to correctly configure them. Although CMMs are accurate, some industries need a faster and easier method to evaluate complex objects.

This accuracy is usually achieved through a Geometric Dimensioning and Tolerancing (GD&T) system. It is a characterization of the object traits, such as straightness, angularity, parallelism, size, that manufactured objects must meet in order to be considered valid. Alternatively, a free-form surface analysis such as the one showed in Pathak et al. [12] can be used, but often a complete definition and evaluation of these traits is complex, time consuming and can barely account for basic defects such as missing or extra material due to small errors in the manufacturing process. Besides this, depending on the acquisition system, retrieved 3D objects may contain synthetic errors that need to be accounted for when evaluating the measurements, making difficult to change the acquisition system without updating the evaluation method. Additionally, some objects are prone to elastic deformations that may not be a defect, for instance springs, making even harder to specify and evaluate through a GD&T model. Finally, a new design requires the specification and validation of the traits by skilled engineers.

There is a large amount of work in 3D object comparison due to 3D content-based retrieval research, see Tangelder et al. [13] for a review. Different techniques are used, such as feature-based methods [14,15,16], graph-based methods [17,18] or more recent deep-learning-based methods [19,20]. Unfortunately, most of this work is focused in pattern recognition and matching, instead of focusing on detecting differences or where these differences are, which is mandatory in most industrial inspection tasks. Additionally, in most cases these approaches require defective pieces that are not available in industrial inspection context. Furthermore, these approaches do not consider objects with manufacturing tolerances.

Other approaches aim to directly compare two 3D surfaces and establish a metric of similarity (or dissimilarity) without specific knowledge of the object traits. See Lague et al. [21] for a review. It is sometimes referred as geometric method [22]. A naive approach could simply be measuring the 3D distance of each point of the evaluated 3D reconstruction to the closest point of the reference (Hausdorff distance). This kind of measures have the advantage that they do not require specification, are easy to implement and describe where the objects differ. It is the kind of approach used in some fields such as geomorphic change detection [23,24,25]. However, they lack the flexibility to correctly measure objects with elastic manufacturing tolerances. This is the main reason why deformable models are preferred instead of the direct use of a similarity metric.

In order to be able to easily evaluate a 3D surface, an analysis involving a statistical shape model (SSM), like the one described in Davies et al. [26], is proposed. To do it, an offline process is performed with a set of human inspected valid objects, obtaining an SSM model that will contain the real variability of the collection of manufactured objects. These models, initially introduced by Mardia et al. [27], gather shape statistics information efficiently in a training stage so the different shape configurations are learned. They are widely used in medical imaging, consult Heimann et al. [28] for a review, mainly focused on aiding segmentation for diagnosis and were recently combined with deep learning strategies in Ambellan et al. [29] or Avendi et al. [30]. Besides medical imaging, SSMs have also been used recently in other fields such as automatic generation of 3D faces in Booth et al. [31] or biology, in order to learn from animal poses, in Zuffi et al. [32].

These SSM models can be used to find abnormalities or differences in samples. In Dall’asta et al. [33] a similar approach to the proposed here is presented. A SSM is built to find abnormalities in fetal facial morphology through a dissimilarity metric based on Mahalanobis distance. The main difference with the presented work is the process is mostly manual, requiring expert knowledge for segmentation, alignment and interpretation of differences. Furthermore, only 20 samples are used. Bruse et al. [34] work is focused on aortic arch shape abnormalities. The shape features present in the SSM built are directly related to pathologies through expert knowledge instead of using a dissimilarity metric. All these approaches are interesting but are focused on solving a single problem using expert knowledge instead of finding significant differences without prior knowledge of the problem. Furthermore, due to the nature of medical data, the datasets used consist of a few tens of samples with a limited amount of points, while industrial inspection operates with thousands or millions of pieces with hundreds of thousands points each. More generic approaches exist such as Erus et al. [35]. In this work, SSMs are used to find abnormalities in brain anatomy without prior knowledge about the characteristics of these abnormalities. In it, an iterative approach is introduced for sampling subspaces and estimate the probability density function of each subspace in a reduced dimensionality space where the test sample is projected. Unfortunately, these kind of approaches are too computationally expensive to be adopted in an industrial inspection pipeline that needs to inspect multiple pieces per second.

To the best of the authors knowledge, SSMs are not used in the context of industrial inspection. This work adapts the use of SSMs in the industrial inspection field in order to be able to correctly evaluate objects with elastic manufacturing tolerances achieving higher accuracy than current practice in many industrial processes. A complete workflow from acquisition to evaluation is proposed in two stages: an offline training stage described in Section 3 and an online evaluation stage presented in Section 4. The main advantages of this method are that it completely avoids human inspection, is able to detect where the surfaces differ, and it only requires valid objects to train the model. Making use of this information, it can evaluate if an inspected object is within the tolerances seen in the training set or not.

## 3. Statistical Shape Models: Training

Training an SSM for industrial inspection is not straightforward. Industrial objects usually require high precision, which involves larger models with a massive amount of points. Additionally, in-line inspection processes must be fast to be able to deal with the high production rates that are common in industry, often equal or higher than one object per second. For these reasons, the training stage must produce an efficient model from very dense samples capable of being evaluated efficiently and with high precision. Fortunately, the training process is typically offline and can take as long as required.

In our case, in addition, the ZG3D device performs a reconstruction from silhouettes of an object in free fall in an undetermined position that produces synthetic bulges which, in the best case, approach the visual hull of the 3D object. This means that different captures of the same object will have slightly different 3D shapes due to the bulges being in different places, according to the different relative positions of the camera views. As a consequence, the learned SSM will also account for these synthetic bulges as variability of the reconstructed object. For instance, training a large object with a narrow hole that can only be seen if a camera is parallel to it will lead to a model with high variability where the hole is.

Usually, SSM training makes use of a set of observations that is as large as possible, to be representative of the variability of the task. However, in this context, each observation contains hundreds of thousands of points, making difficult to process all of them at the same time. Taking this into account, an iterative strategy has been followed—the observation is aligned with the current model, then the model is updated with the object information to produce the SSM model for the next iteration. This can be done until the model converges, a possible convergence criterion is that the means and variances converge to a stable value. In the first iteration, the observation is directly copied to the model, so it will be as dense as the first sample.

This strategy makes impossible the dimensionality reduction used in the training set by some authors. Fortunately, in industrial inspection it is easier to control that the training samples used are valid and effectively represent the range of possible input surfaces to the system. Therefore, it is assumed that the training set has been manually checked, it is correct, and does not contain a significant amount of outliers that could lead to an incorrect model estimation.

The SSM model will be formed by a set of points with as much information as required, but at least the 3D position. Each point will be assumed to follow a multivariate normal distribution (multidimensional Gaussian), so the only information required is the mean and covariance matrix. Additionally, the points will keep the edge relation information (set of neighbours) that allows building a 3D mesh, and which will be used for the final smoothing and representation step.

**Figure 2:**
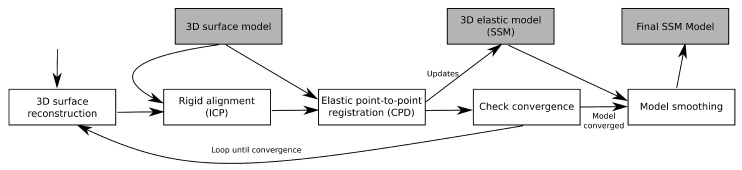
Statistical Shape Model(SSM) Training diagram, that describes the process.

A general diagram of the training process is shown in Figure 2. The procedures can be seen in the white boxes while the inputs and generated outputs are represented as grey boxes. Although well-known algorithms are suggested for some of them, other alternatives may be used without loss of generality.

The first step to train an SSM model is the alignment. The object to be added to the model needs to be aligned with it. First a rigid alignment in order to place object and model in the same position is performed. Many different solutions exists, in this case a rigid Iterative Closest Point (ICP) registration is performed as described in Kok-Lim [36], so both, model and observed object are aligned. At this point, the distance from every point of the object to the model is minimized allowing to move and rotate the complete object rigidly.

**Figure 3:**
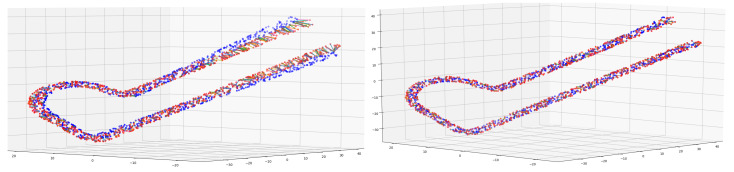
Elastic alignment example. Left image: rigidly aligned point clouds, right image: point clouds elastically aligned. Red points correspond to model while blue is a new sample to train.

However, it is necessary to register every point in the object with the corresponding point in the model. As can be seen in left image of Figure 3, even after a correct rigid alignment point correspondence is not trivial due to elastic differences between objects, and closest neighbor does not provide a correct solution in this cases. So an elastic point-to-point registration is required. In order to do so, a non rigid alignment that maps all the points of the object to the model is performed. Castellani et al. [37] can be consulted for a review of different methods. In this case the State of the Art Coherent Point Drift (CPD) [38] has been used. Once the surfaces are elastically aligned as the example shown on the right image of Figure 3, point-to-point correspondence can be trivially solved pairing closest points of object and model surfaces.

Once this registration has been achieved, the information of each pair of points is accumulated in the mean and covariance matrices model, keeping track of the number of pairs which information is already accumulated in each model point. In this case, the most relevant information is the 3D point position, but the normal vector associated to the surface at that position is also relevant as shape information, and depending on the type of object, other characteristics, such as the color, could also be relevant. Any information associated to the points could be added to the model in a similar way. The results shown in this work include the position-only and the position+normals versions to show the importance of the normal vectors in the shape comparison process.

The model is trained with new correct objects from the available set, checking the convergence of the 3D point positions until the means have converged below a predefined threshold. A simple strategy to establish this threshold is to choose an order of magnitude or two below the resolution of the capturer device, in this case the ZG3D device. This threshold ensures the training stage has finished, as the changes in the model are small in comparison with the system’s precision. The thresholds for other dimensions can be similarly established.

Finally, a model smoothing step is necessary to maintain consistency, specially in cases with limited training sets. At this point, the model being learned is a point cloud where each point *p* contains the accumulated information of all the samples points that where registered with this *p* point. This accumulated information is the means and covariance matrix of selected characteristics which include at least 3D position information and may include surface normal at the point, color...and so forth. However, this information may be non-uniformly gathered, some points may have accumulated characteristics from a few samples while its neighbours may have accumulated hundreds or thousands samples characteristics. This fact may produce a noisy model that must be smoothed.

In order to do so, each point of the model being learned is averaged with its local neighbours by weighting number of accumulated pairs. This process also accounts for a global object accumulated information smoothness: mean and covariance of characteristics should not change abruptly along the model. Equation (1) describes the smoothing process for the statistical information SI of a point *p* where $N_{p}$ is the neighbourhood of *p*, $D_{q}$ is the point density in *q*, which is a point inside the neighbourhood of *p*.

(1) $$SI_{p}=\frac{\sum_{q\in N_{p}}SI_{q}D_{q}}{\sum_{q\in N_{p}}D_{q}}.$$

The parameter $N_{p}$ is a parameter that allows to restrict the smoothing locally. It can be set as a distance threshold or as a geodesic distance in the surface mesh. In this work it has been experimentally set to 50 μm, which is around the maximum system resolution of 1 pixel at working distance. A higher value produces smoother models less prone to small errors caused by poor training, but may ignore small defects.

## 4. Evaluation of 3D Surfaces

In order to evaluate a 3D mesh against the previously trained model, it will be measured the point-to-point difference between 3D surface mesh and model with learned variation. A feasible strategy would be to repeat the rigid and elastic-alignment, used in the train stage, and directly use a metric. However, elastic-alignment is a very time-consuming operation, which is an important factor in industrial inspection. For this reason, only a rigid alignment is performed to initially align both surfaces and speed-up the evaluation process. For this evaluation, a dissimilarity metric is proposed based on surface-model and model-surface distances. The dissimilarity metric includes a smoothing area filter to ignore small-sized errors.

These two distances focus on finding extra and missing volume respectively. The first one will guarantee that every point in the model has at least one point in the reconstructed object closer than a threshold, so all the characteristics in the model are present in the object. The second is the reverse property: every point in the reconstructed object should have a point in the model closer than a threshold, in other words, every reconstructed characteristic of the object has an associated part of the model. Using only the first metric, it would miss samples with extra volume in the reconstructed object, while considering only the second test, it would not recognize the cases where the object has a missing volume error.

Both metrics are computed in the multivariate normal space using Mahalanobis distance. Mahalanobis distance is a metric normalized with the standard deviation, so it measures the distance in standard deviations from observation $\vec{x}$ to a set of observations with mean $\vec{\mu }$ and covariance matrix *S* that can be computed using Equation (2). In practice, assuming a normal distribution of the deformations, a threshold around 4 standard deviations (99.99% confidence), depending on the industrial application, could be reasonable.

(2) $$D_{M}\left(\vec{x}\right)=\sqrt{{\left(\vec{x}-\vec{\mu }\right)}^{T}S^{-1}\left(\vec{x}-\vec{\mu }\right)}.$$

As this metric takes into account the covariance matrix, the same difference with respect to the mean value will result in a smaller error in the measurements that have higher variability and a higher one where the object tolerances are smaller. It is a measure of the likelihood of a given observation to belong to the probability distribution.

Formally, due to the non-euclidean characteristic of the metric, each tested point must be checked against each point in the model in order to get the minimum distance. Unfortunately, this means the algorithm would be in O(mn) where *m*, *n* are the number of points in model and test surfaces. As the application requires fast computation and the precision of the evaluation also depends on the *m* and *n* values, a trade-off has been adopted: only the *k* closest points in euclidean space, that can be found in a bounded period of time, are checked, reducing the problem complexity to find the minimum distance to O((log(m)+k)n) assuming a small error. In the equation, log(m) is the k-neighbours search and *k* accounts for the *k* distances that need to be computed. As *m* is in the order of hundreds thousands then (log(m)+k)<<m effectively speeding up computation. In the experiments k=200 proved to be good trade-off between performance/precision.

Using this metric, it is possible to determine the distance from each point of the reconstructed surface to the model and from each point in the model to the tested surface. Once a distance has been computed for all the points in the surface, a smoothing area filter is applied to find the highest difference between surfaces. The smoothing is required as high outliers on small areas may not represent the real difference between surfaces. A difference value *X* is defined as an area of at least *A* size, where all its forming points have at least *X* distance, thus actually filtering high outliers.

This requires two user-selected parameters size-threshold *A* and error threshold *X*. The size error is the minimum size of an area in the surface in which all contained points must be defective in order to be considered a defective surface. High values ignore errors that may not be relevant or come from acquisition errors. In the experiments, this threshold has been set to 0.2 mm${}^{2}$ which is double the minimum defect in the set. The error threshold is the minimum distance to the model in order to consider a point defective. This threshold allows to change the sensitivity of error detection. It is the changing value for the ROCs in the experiment section, although 4σ is proposed as a 99.99% confidence threshold.

**Figure 4:**
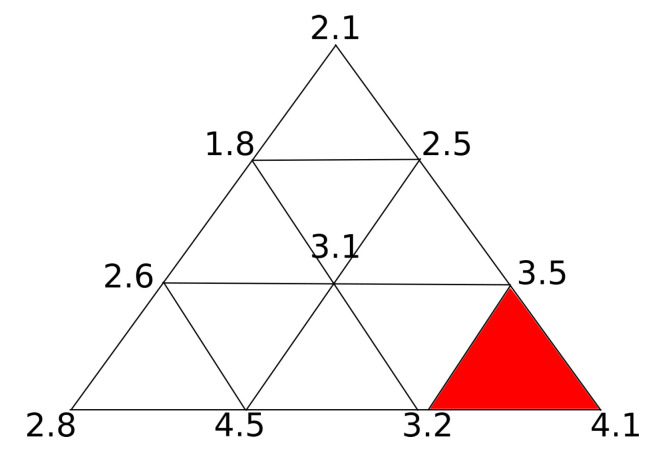
Highest error thresholding example in a surface mesh where each vertex has an error associated and area threshold is set to triangle size. Highlighted in red is the triangle with the highest error with 3.2σ error.

Consequently, the highest difference between surfaces is the highest difference value of all the possible areas of at least *A* size. An example can be seen in Figure 4, where the neighbourhood threshold *A* is set just below the surface triangle size, the global distance would be 3.2 as the only triangle which points are all equal or above is the one highlighted in red, ignoring points with higher error. Although the example area limit is exactly one triangle, *A* is not restricted to triangles, as they may be of different sizes, and a valid area may include more than one triangle. This process ends with two measurements: the distance from the model to the reconstructed object and the distance from the reconstructed object to the model.

Finally, the evaluation score, that measures the difference between two surfaces, is the maximum of these measurements. This value will be the final difference measured in standard deviations between the model and reconstructed object, and vice-versa.

## 5. Methodology Assessment

In order to compare the proposed method with different algorithms, two additional evaluation methods commonly used have been tested. Additionally, we considered two variants of our SSM-based algorithm: using only 3D point information (SSM-Points), and including the normal vector (SSM). In the experiments in this section, each surface has been evaluated with the three algorithms. In the case of trainable algorithms, the training set used was the same.

- Cloud to Cloud (C2C) comparison: This algorithm measures the distance from each point of one surface to the other as a Hausdorff distance. The implemented version makes both measurements from each point of the evaluated surface to the model surface and vice-versa, so the object differences are not missed. In this case, the model is a representation of the expected object. In order to be considered a defective object, the error must be larger than a predefined threshold, so the errors are filtered according to the neighbourhood information. The final evaluation of the object will be the highest distance of at least the minimum size established. This is performed for both evaluations—test surface against model and vice versa. The main disadvantage of this method is that it does not learn the variability along the object, on the other hand it is easy to implement, it does not require training and it is fast to compute, which is important in an industrial environment.
- Kernel-hull (KH) volume difference: This algorithm is an extension of the C2C comparison. In order to learn the variability, two models are created—kernel and hull. The kernel is the volume that is common to all the training objects, while the hull is the volume that was present in at least one object of the training set. In order to compute these models, each object must be reconstructed and aligned, and after that, an intersection operation is performed with the current kernel to update it. Then, a union operation gives rise to the final hull. The evaluation in this case entails obtaining the missing volume of the test object with respect to the kernel, and the extra volume with respect to the hull. This method is able to learn some variability and is moderately fast to compute.

## 6. Results

In order to compare the performance of the three algorithms, an ROC curve has been depicted in each experiment, expressing the performance changing the free parameter distance threshold while keeping a constant size threshold. In this case, the true positives are the defective objects labelled as invalid while the false positives are the correct objects labelled as not valid.

In the case of industrial inspection it is of utmost importance to detect all defective pieces. This is because a single false negative could cause a high economical loss, for instance it could cause a car crash, while discarding a few false positives has a low economical impact, as evaluated pieces are cheap to produce. Taking this into account, Precision at Total Recall (PaTR) will be considered the key indicator to compare algorithms, although complete ROC curve is shown as it may be interesting in other application fields. This is the minimum value of False Positives rate, *X* axis, where True Positives rate, *Y* axis, arrives 1.0.

A first analysis has been performed in a simulation environment and then another set of experiments has been conducted with the real device.

### 6.1. Results with Synthetic Data

Different views of an object were synthetically generated and submitted to the 3D reconstruction process that is used in a ZG3D device. This allows us to also generate the synthetic errors produced by reconstruction from silhouettes. Furthermore, the position of the 3D object with respect to the model is known, so the alignment object of the algorithm can be ignored to directly compare the metrics.

**Figure 5:**
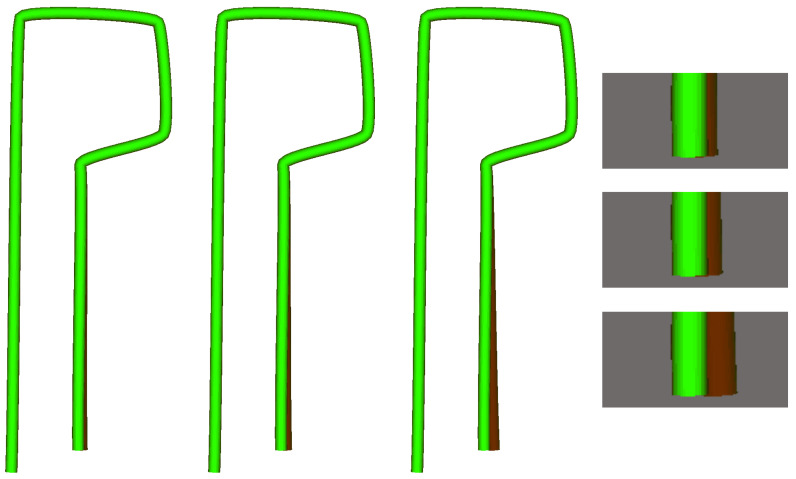
Synthetic wire figures used for experimentation. The bright green surface is the perfect (average) model, while the dark red areas illustrate a test object with increasingly larger deformations.

A number of 3D objects were automatically created by modifying the control points of a sequence of vertices forming a bezier curve that were then converted to 3D surfaces by means of extruding a circle along the curve. With this simple technique, wire figures as the ones in Figure 5 can be easily created. A movement tolerance in each axis has been established for each point, so the wired figure can change its shape differently in each axis and vertex. Controlling the movement of the control points, four different sets of surfaces were created:

- Reference set: 500 surfaces generated with random noise within the tolerances of each control point. This set will be used exclusively for the training step.
- Good Validation set: 100 surfaces generated identically as the previous set, but using a different random seed, so the final surfaces are different.
- Incorrect Validation set: 100 surfaces in which one of the control points has moved 3× times the tolerance, and the rest have random noise inside the tolerance range.
- Incorrect Difficult Validation set: Similar to the incorrect set but the erroneous control point has moved 1.5× times the tolerance. That gives rise to an error below 2 pixels that is close to the simulated hardware resolution limit.

Each synthetic surface in the Validation sets has been tested 10 times simulating random positions and rotations in the ZG3D-device to make it more realistic. Each surface in the Reference set is also randomized so the algorithms can learn the variability produced by the device used. However, in this first experiment the object alignment has been synthetically corrected in order to isolate the evaluation results from possible alignment errors.

**Figure 6:**
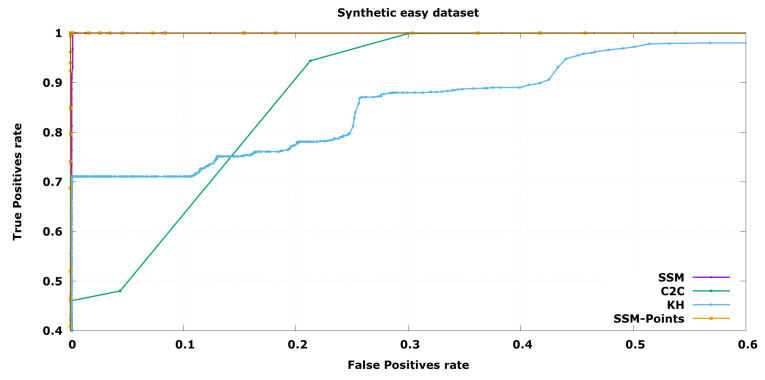
ROC curve for synthetic object classification from the Good and Incorrect validation sets synthetically aligned.

In Figure 6 the ROC curves for the task of classification between Good and Incorrect objects are presented. Following the bibliography naming convention, a defective object is a “positive” result. The results show that the proposed SSM-based solutions, SSM and SSM-Points, are capable of achieving almost perfect discrimination of results while the alternative algorithms fail in some cases. C2C evaluation is unable to find errors in control points with small variability without discarding correct objects that have a high value in the high variability control points. This is the main problem of having a constant threshold through all the object. On the other hand KH algorithm is not capable of distinguishing incorrect objects when they are smaller because the defective object is already discarded from the kernel due to high variability of the training samples, this makes the algorithm not adequate for most wire-shaped objects.

**Figure 7:**
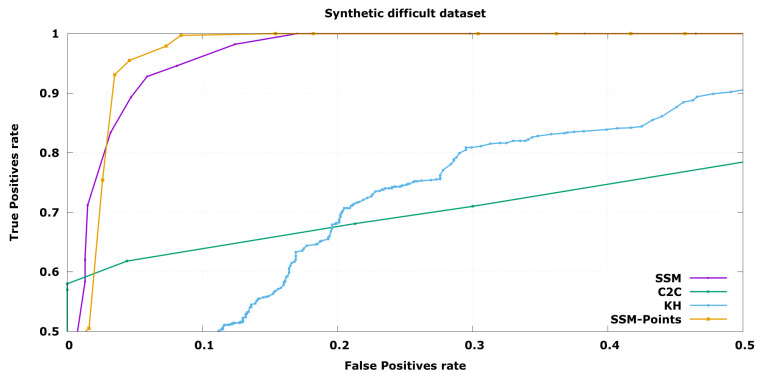
ROC curve for synthetic object classification between the Good and Difficult validation sets synthetically aligned.

When a more challenging set is used, the difference among the algorithms increase, as can be seen in Figure 7. Even in this case, the probabilistic comparison is capable of producing reasonable results. This means that the objects that are almost within the established threshold will be correctly detected, only discarding below 10% of the correct set it is able to find all the defective pieces, while the other approaches need to discard beyond 50%.

In this case, differences between the two SSM algorithms can be seen. Regarding PaTR, which is the preferred working point for industrial inspection, the points and normals results are better. Nevertheless, SSM with only points information obtains similar results in most working points because it ignores noisy inputs and may work better depending on the application.

In both cases the optimal threshold is around 4 standard deviations which is a correct value assuming the normal distribution as it means 99.99% of the samples are inside of it. Thus it proves the trained model was able to learn the synthetic data correctly.

These results show that in the case of wired-shaped objects with elastic manufacturing tolerances, the SSM-based solutions obtain significantly better results. This method does not need specific knowledge of the problem as other trait-based solutions that require object-dependent characterization of the variability. Furthermore, this solution only requires valid objects to infer a model that is capable of rejecting objects that are different from the Reference set.

### 6.2. Results with Real Data

**Figure 8:**
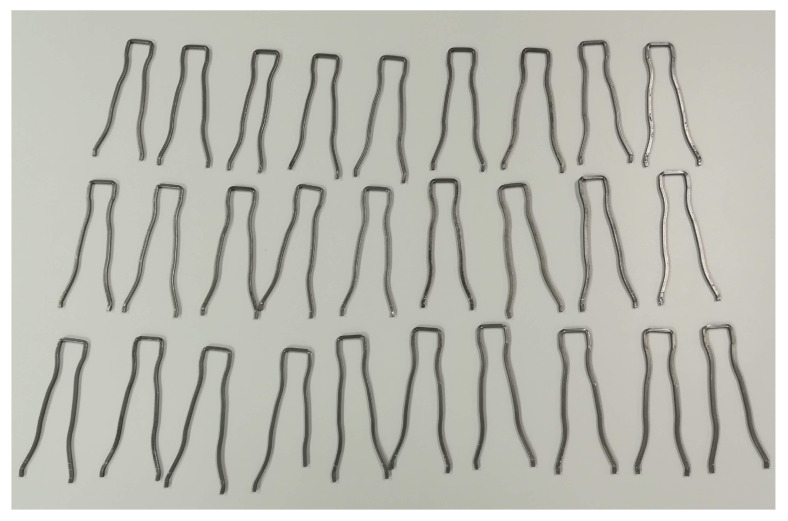
Set of objects used for the experiments with real data.

To complement and validate the simulated results, additional experiments have been carried out in the real inspection device. In this case, the set of objects have been limited to assure correct labelling of the dataset, however the number of repetitions have been increased so the results are statistically relevant. Furthermore, it is difficult to establish a challenging dataset where the defective objects are close enough to the correct samples. For these reasons, the dataset is smaller as can be seen in Figure 8. Each of the objects has been inspected 25 times, so the variability of the device is also modelled during the process.

The main difference with the synthetic dataset is that the Good Validation set models have small tolerances, this makes the results of the methods more similar. As a consequence, it is a more challenging test for the proposed method that expects elastic manufacturing tolerances, which in this case will be product of errors during the process or synthetic bulges due to the silhouette reconstruction methodology in most cases. Even in this situation, the proposed method could outperform the alternatives due to its use of local information which allows it to learn this variability at a fine-grained level.

**Figure 9:**
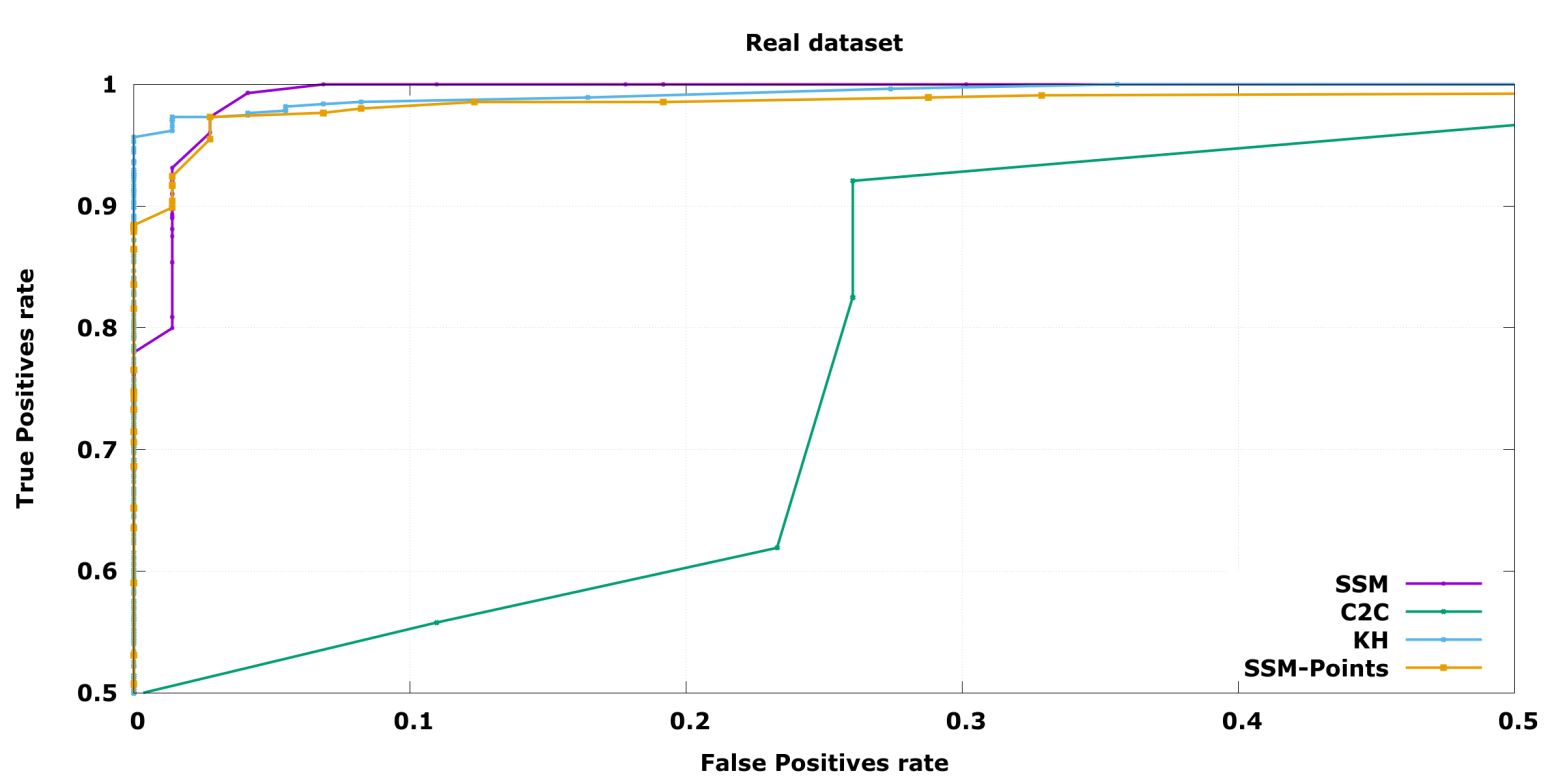
ROC curve for real objects classification (the objects in this set are barely distinguishable to the naked eye).

The results in Figure 9 show that the proposed method is significantly better in the most important range of the ROC graph for industrial inspection operations. It is capable of virtually 100% detection of defective objects accepting 93.15% of the non-defective objects, while the kernel-hull method just accepts 64.38%. The SSM only with the position information shows a lower performance, and the results of C2C are far from the other algorithms.

It is important to note that this dataset has been selected to have a larger than normal proportion of defective objects, and objects with very small errors, close to the limit of acceptance. This gives rise to results that are significantly worse than those found be in a real industrial process, where it is expected that most of the objects are correct. It is also possible to design an inspection process where the objects that are found to be close to the acceptance limit are analysed a second time to increase the precision.

**Figure 10:**
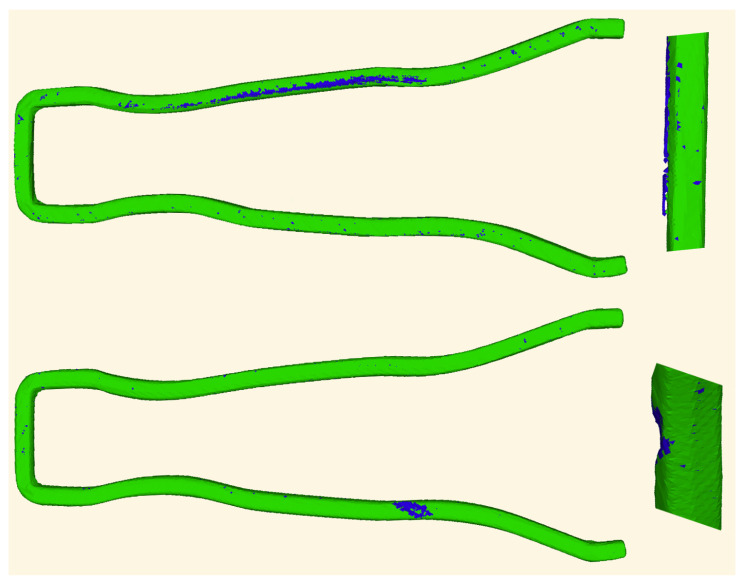
Top image: typical correct object incorrectly classified by SSM. Bottom image: typical defective object not detected by KH.

Two representative errors that help to characterize the kernel-hull and SSM algorithms are shown in Figure 10. The bottom image shows an object with missing material that the SSM has correctly labelled (green is correct and blue incorrect). However, the kernel-hull algorithm was not capable of detecting the error because the missing material is too thin and is within the variability of the set of reference objects. For this reason, SSM was only able to detect it by using the information of the local normal vectors. Although the 3D position could be correct according to the variability, the normals showed large changes that made it different from the reference objects.

The top image shows a correct object with unusually large synthetic bulges caused by the silhouette 3D reconstruction. These bulges often behave as outliers that do not follow a Gaussian distribution and are thus not learned during the training stage. Therefore, SSM incorrectly detects them as erroneous. The kernel-hull technique follows a maximum-minimum strategy that ignores the distribution, so in this case it does not detect this bulges as erroneous. The position and size of these bulges can be predicted by taking into account the camera distribution and the shape of the objects. That information could be combined with the SSM estimated Gaussians to avoid these kind of errors, but this is out of the scope of this paper and is proposed as future work.

The results of this experiment show that the method presented outperforms other solutions even in cases where the deformations are small and of rigid nature, where it should have similar performance. The availability of local statistical information of the point positions and their normal vectors to the surface are key to this advantage. Although the kernel-hull comparison is slightly better in some operating areas of the ROC curve, the statistical shape model based evaluation is significantly better detecting all the defective samples at a higher rate of true positives which is the desired working point for industrial inspection.

## 7. Conclusions

A probabilistic method for training models and evaluating 3D surfaces with elastic manufacturing tolerances has been presented. The training process makes use of correct objects to infer a model shape with variability, making possible to measure distances with the learned Statistical Shape Model (SSM). The evaluation relies on it to analyse the 3D reconstructed object establishing a metric for each point against this model. This evaluation may be filtered against outliers to determine whether the inspected object is correct or not.

The proposed method has been compared with other alternatives in simulated and real environments using a ZG3D device, outperforming them for the industrial inspection use case. Furthermore, the SSM based algorithm proved to be capable to work with rigid as well as elastic manufacturing tolerances, obtaining better results than the alternative algorithms in both cases.

The main source of errors is the non-Gaussian distribution of synthetic bulges caused by the silhouette reconstructions when the object is captured from different angles. As future work, these errors can be fixed ignoring these bulges, as their locations and shapes only depend on the position of the object with respect to the cameras, which can be estimated.

## Abbreviations

The following abbreviations are used in this manuscript:

SSM	Statistical Shape Model
ZG3D	Zero Gravity 3D
GD&T	Geometric Dimensioning and Tolerancing
ICP	Iterative Closest Point
CPD	Coherent Point Drift
C2C	Cloud to Cloud
KH	Kernel Hull
ROC	Receiver Operating Characteristic
